# Comparative Physical Study of Three Pharmaceutically Active Benzodiazepine Derivatives: Crystalline versus Amorphous State and Crystallization Tendency

**DOI:** 10.1021/acs.molpharmaceut.1c00081

**Published:** 2021-03-09

**Authors:** Sofia Valenti, Maria Barrio, Philippe Negrier, Michela Romanini, Roberto Macovez, Josep-Lluis Tamarit

**Affiliations:** † Grup de Caracterització de Materials, Departament de Física and Barcelona Research Center in Multiscale Science and Engineering, Universitat Politècnica de Catalunya, EEBE, Campus Diagonal-Besòs, Av. Eduard Maristany 10-14, Barcelona, Catalonia 08019, Spain; ‡ Université Bordeaux, Laboratoire Ondes et Matière d’Aquitaine, UMR 5798, 351 Cours de la Libération, Talence F-33400, France

**Keywords:** Valium, crystal structure, Hirshfeld analysis, dielectric relaxation, glass transition, hydrogen bonding, ring inversion, crystallization kinetics, Avrami law, physical stability

## Abstract

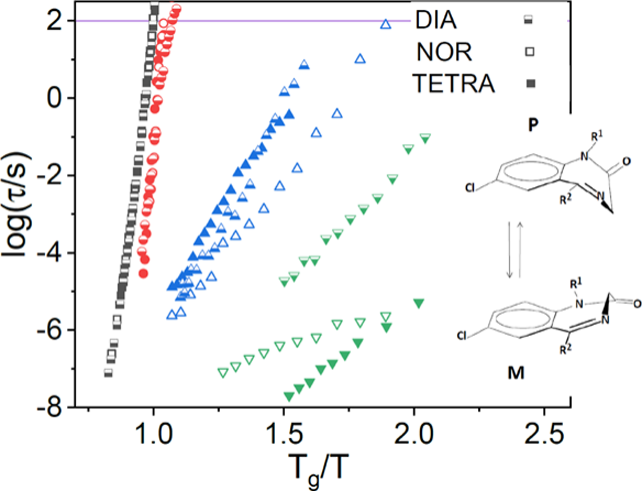

Chemical derivatization and amorphization are two possible strategies to improve the solubility and bioavailability of drugs, which is a key issue for the pharmaceutical industry. In this contribution, we explore whether both strategies can be combined by studying how small differences in the molecular structure of three related pharmaceutical compounds affect their crystalline structure and melting point (*T*_m_), the relaxation dynamics in the amorphous phase, and the glass transition temperature (*T*_g_), as well as the tendency toward recrystallization. Three benzodiazepine derivatives of almost same molecular mass and structure (Diazepam, Nordazepam and Tetrazepam) were chosen as model compounds. Nordazepam is the only one that displays N–H···O hydrogen bonds both in crystalline and amorphous phases, which leads to a significantly higher *T*_m_ (by 70–80 K) and *T*_g_ (by 30–40 K) compared to those of Tetrazepam and Diazepam (which display similar values of characteristic temperatures). The relaxation dynamics in the amorphous phase, as determined experimentally using broadband dielectric spectroscopy, is dominated by a structural relaxation and a Johari–Goldstein secondary relaxation, both of which scale with the reduced temperature *T*/*T*_g_. The kinetic fragility index is very low and virtually the same (*m*_p_ ≈ 32) in all three compounds. Two more secondary relaxations are observed in the glass state: the slower of the two has virtually the same relaxation time and activation energy in all three compounds, and is assigned to the inter-enantiomer conversion dynamics of the flexible diazepine heterocycle between isoenergetic P and M conformations. We tentatively assign the fastest secondary relaxation, present only in Diazepam and Tetrazepam, to the rigid rotation of the fused diazepine–benzene double ring relative to the six-membered carbon ring. Such motion appears to be largely hindered in glassy Nordazepam, possibly due to the presence of the hydrogen bonds. Supercooled liquid Tetrazepam and Nordazepam are observed to crystallize into their stable crystalline form with an Avrami exponent close to unity indicating unidimensional growth with only sporadic nucleation, which allows a direct assessment of the crystal growth rate. Despite the very similar growth mode, the two derivatives exhibit very different kinetics for a fixed value of the reduced temperature and thus of the structural relaxation time, with Nordazepam displaying slower growth kinetics. Diazepam does not instead display any tendency toward recrystallization over short periods of time (even close to *T*_m_). Both these observations in three very similar diazepine derivatives provide direct evidence that the kinetics of recrystallization of amorphous pharmaceuticals is not a universal function, at least in the supercooled liquid phase, of the structural or the conformational relaxation dynamics, and it is not simply correlated with related parameters such as the kinetic fragility or activation barrier of the structural relaxation. Only the crystal growth rate, and not the nucleation rate, shows a correlation with the presence or absence of hydrogen bonding.

## 1 Introduction

The chemical modification of active pharmaceutical ingredients (APIs) is one of the main strategies to identify better drugs with reduced side effects and increased efficacy or bioavailability. A historical example is that of the active ingredient of aspirin: derivatization of salicylic acid, the active principle present in willow barks, into acetylsalicylic acid leads to substantial reduction of the side effects of the naturally occurring drug.^1^ Given that low solubility in water and thus low oral bioavailability is one of the main issues in current drug research, chemical derivatization of APIs in the form, e.g., of hydrochloride salts with enhanced solubility is often pursued.^2,3^ Another related strategy for efficient drug administration is the development of a prodrug, i.e., an inactive compound (usually a derivative of an active drug) that undergoes *in vivo* transformation, through enzymes or metabolic processes, into the active parent drug. This strategy has been applied successfully to improve the pharmacokinetic properties of drugs since the middle of the last century, when the term prodrug was first introduced.^4^ Nowadays, prodrugs make almost 10% of the administered drugs, reaching a peak of 20% of the market between 2000 and 2008.^5,6^

While chemical derivatization is mainly aimed at identifying drugs with better biochemical properties, it also obviously affects the physical properties of the parent API. In the vast majority of cases, the induced changes in physical properties stem from relatively minor chemical changes, as the derivative (prodrug, salt, etc.) is usually one or two metabolic steps away from the active parent drug.^7^ The chemical modification may, for example, determine a modified crystal structure of the resulting drug and have an impact also on the possible polymorphism and relative stability of different crystalline forms, which is of relevance for API storage prior to industrial processing. These aspects are extremely important for the pharmaceutical industry, as polymorphism or the possible stability of an amorphous (glass and supercooled liquid) phase can have a strong impact on the viable protocols for the preparation of suitable formulations for the administration of APIs.^8,9^

Drug derivatization also affects the glass transition temperature and the kinetic stability of the amorphous form of the drug. It is well-known that amorphous pharmaceuticals have better dissolution and thus better bioavailability properties than their crystalline counterparts,^10,11^ and a few amorphous drugs have appeared on the market in recent years.^12,13^ The amorphous form of a drug may be present in a formulation as a result of industrial processing via, e.g., milling and spray or freeze drying.^14−16^ Despite their advantage in terms of solubility, however, amorphous drugs are not thermodynamically stable and are thus prone to recrystallization into the lower-solubility crystalline form.^9,17−19^ A better understanding of the amorphous state is needed to advance in the formulation of amorphous drugs. In the context of drug modification strategies, it would be extremely useful to be able to predict how different drug derivatives behave in terms of kinetic stability and tendency toward recrystallization of the amorphous form, both in the case of amorphous API phases formed spontaneously or purposefully during formulation of a medicament. The present paper takes a step in this direction by comparing the physical properties of the amorphous and crystalline forms of three distinct pharmacologically active benzodiazepines, with the aim of exploring possible routes to increase the kinetic stability of amorphous derivatives.

**Figure 1:**
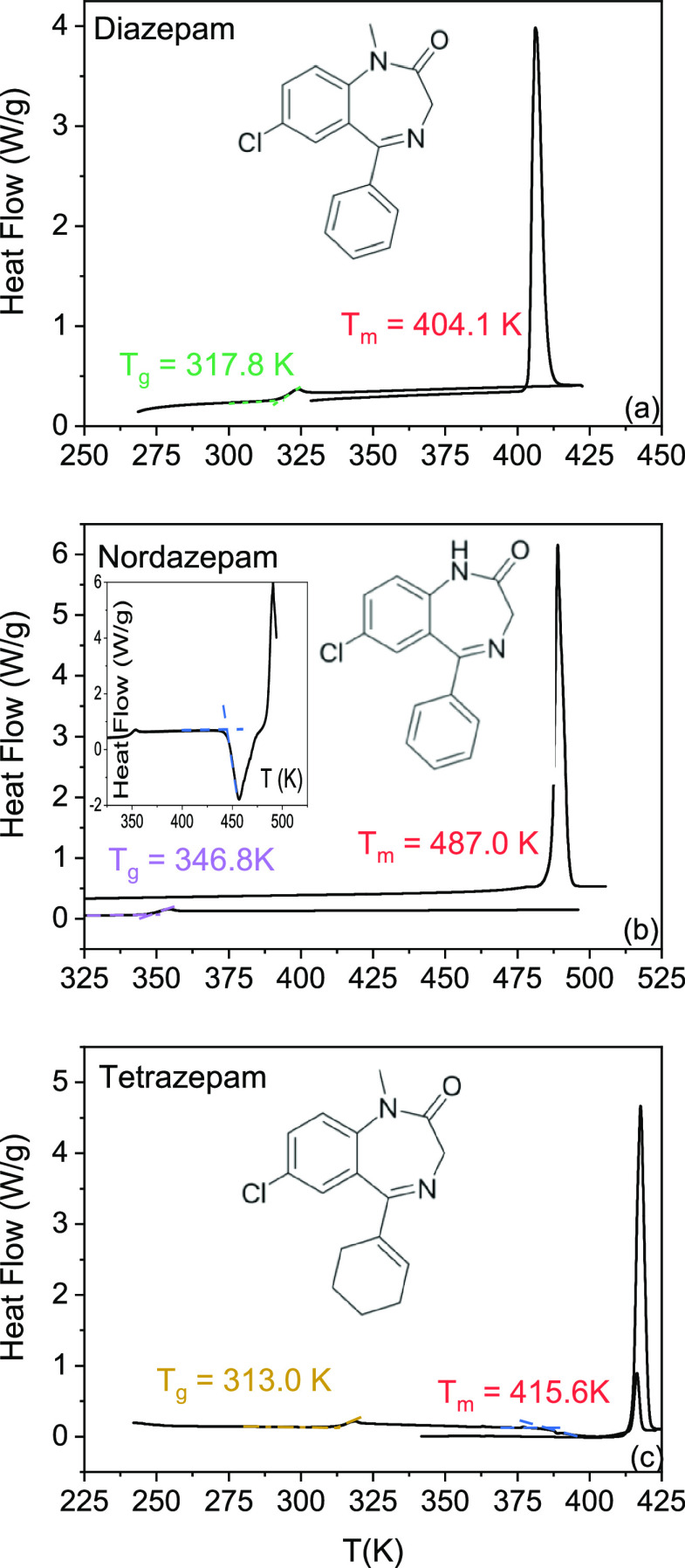
DSC traces of DIA (a), NOR (b), and TETRA (c) obtained with a 10 K min^–1^ heating/cooling rate. Upward peaks are endothermic processes. Inset to (b): a different heating ramp acquired on amorphous NOR, where recrystallization is visible. The experimental determination of the glass transition, recrystallization, and melting temperatures are indicated with dashed lines.

The common molecular structure of the benzodiazepine drugs consists of a rigid benzene ring and a flexible diazepine ring fused together. Several benzodiazepines also display a third six-membered ring covalently attached to a carbon atom of the diazepine ring (see, e.g., the molecular structures displayed as insets to Figure 1). These drugs work by enhancing the effect of the gamma*-*aminobutyric acid neurotransmitter, and they have sedative, hypnotic, anxiolytic, anticonvulsant, and muscle relaxant properties. According to a WHO report of 2017, 322 million people suffer from depression as of 2015 and almost as many suffer from other anxiety disorders^20^ and it is estimated that 40% of patients with depressive and anxiety disorders are prescribed benzodiazepines.^21^ Oral administration is the most common route of administration of benzodiazepines (although injectable, inhalation, and rectal forms are also available), but, given that they are lipophilic drugs, problems of low solubility and bioavailability may arise in the gastrointestinal tract.^22,23^ Low bioavailability may result in the need of a higher dose administered to the patient, to account for the percentage that is not absorbed and metabolized. This may lead to undesirable adverse side effects, which are already pretty severe with high doses of this type of drugs.

Here, we study three related benzodiazepine derivatives: Diazepam, Nordazepam (also known as Nordiazepam or desmethyldiazepam), and Tetrazepam. Diazepam (see inset to Figure 1a) is one of the best known benzodiazepines and was first marketed as Valium. It is used as a treatment for various mental diseases, but its primary use is for anxiety, states of agitation, or panic attacks. Diazepam has been studied extensively in both crystalline and amorphous states, sometimes in comparative studies with other benzodiazepines.^24−27^ Its main active metabolite is Nordazepam, whose chemical structure differs from that of Diazepam only by the substitution of the methyl group linked to the nitrogen 1 of the diazepine by a hydrogen atom (see the inset to Figure 1b). This difference, however, is highly significant in that it confers the Nordazepam derivative the possibility of self-aggregation via hydrogen bonding via the H-functionalization of the nitrogen atom. Tetrazepam (inset to Figure 1c) differs from Diazepam in that the benzene ring attached to the carbon 5 of the diazepine ring is substituted by a cyclohexene ring. It was marketed principally as a treatment for muscle spasms and panic attacks but was suspended from the market across the European Union in 2013, due to cutaneous toxicity.

Our comparative study of these three pharmaceutically active ingredients encompasses both their crystalline and amorphous forms (supercooled liquid and glass), as well as the transition between the supercooled liquid phase to the crystalline one. We focus in particular on the molecular conformations and intermolecular interactions in the crystal phase, Hirshfeld surfaces, calorimetric properties, dynamic relaxations, and recrystallization kinetics, the latter two measured by dielectric spectroscopy. Our aim is to understand how the modifications in molecular structure and the resulting intermolecular interactions affect the crystal structure and molecular dynamics in the amorphous phase, as well as the melting point, glass transition temperature, and tendency toward recrystallization of the various derivatives, with the aim of identifying possible structure–property correlations. The study of molecular relaxation processes in diazepines is particularly interesting due to the inherent flexibility of the seven-membered diazepine ring, which leads to conformational diversity of the molecules and therefore to the possible existence of a relaxational inter-conformer conversion dynamics. To the best of our knowledge, only a few very recent studies have focused on the interpretation of the dielectric relaxation of flexible heterocyclic molecules.^28^ A further outcome of this work is therefore to expand the current experimental knowledge of the conformational dynamics of flexible cyclic or ring-containing molecules.

## 2 Materials and Methods

Tetrazepam (TETRA, hereinafter) is a powder of medicinal grade kindly supplied by Daiichi Sankyo France SAS. Samples of medicinal grade Nordazepam (NOR) were kindly provided by Bouchara-Recordati (France) and medicinal grade Diazepam (DIA) was kindly supplied by Neuraxpharm (Spain). The powders of the three diazepines, with purities higher than 99.5%, were used as received without further purification. Differential scanning calorimetry (DSC) experiments were carried out under a nitrogen atmosphere on samples loaded in pierced aluminum pans, by means of a Q100 calorimeter from TA Instruments. Measurements were performed using heating/cooling rates of 10 K min^–1^ and sample masses of the order of 5 mg, as determined with a microbalance with 0.01 mg sensitivity.

Powder X-ray diffraction patterns have been acquired by means of a vertically mounted INEL cylindrical position-sensitive detector (CPS-120) using the Debye–Scherrer geometry and transmission mode. Monochromatic Cu Kα_1_ (λ = 1.54056 Å) radiation was selected by means of a quartz monochromator. Cubic phase Na_2_Ca_3_Al_2_F_4_ was used for external calibration. The analysis of the diffraction patterns (fitting of diffraction peaks by means of the Materials Studio software^29^) was carried out using the published monoclinic (P2_1_/c) structures of TETRA,^30^ DIA,^31^ and NOR.^32^ Hirshfeld surface analyses were performed by means of the CrystalExplorer software (https://crystalexplorer.scb.uwa.edu.au/).

Broadband dielectric spectroscopy (BDS) measurements were carried on the amorphous form (supercooled liquid and glass states) of the drugs, by means of a Novocontrol Alpha analyzer. The samples were placed in a stainless steel parallel-plate capacitor specially designed for the analysis of liquid samples, with the two electrodes kept at a fixed distance by means of cylindrical silica spacers of 50 μm diameter. Temperature control of the capacitor and thus of the sample was achieved with a nitrogen-gas flow cryostat with a precision of 0.1 K. To obtain the amorphous form, the powders were initially melted in the capacitor outside the cryostat, cooled at room temperature, and melted again inside the cryostat. Each sample was then cooled with a cooling rate of 10 K min^–1^ to 123 K to avoid recrystallization, and isothermal spectra were then acquired every 2 or 5 K, waiting each time 5 min for temperature stabilization. Dielectric spectra were measured in the frequency range between 10^–2^ and 10^7^ Hz, from 123 K up to the melting temperature of each compound (404.1, 415.6, and 487 K, for Diazepam, Tetrazepam, and Nordazepam, respectively).

To obtain relaxation times and quantify the changes in relaxation dynamics, we employed the Grafity software to fit the dielectric spectra as the sum of a power law representing the dc conductivity contribution, modeled as a term of the form  in the complex permittivity, where *s* is an exponent close to unity, and a Havriliak–Negami (HN) function for each relaxation component.^33^ Overall, the spectra contained four different relaxation components (referred to as α, β, γ, and γ′ in the text), and the total complex permittivity was modeled as follows:
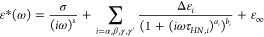
1Here, ω = 2πν is the angular frequency, ε_∞_ is the permittivity in the high frequency limit, Δε*_i_* is the dielectric intensity (or relaxation strength) of relaxation *i* (*i* = α, β, γ or γ′), *a_i_* and *b_i_* are parameters describing the shape of the corresponding loss curves, and τ_HN,*i*_ is a time parameter connected to the characteristic relaxation time τ_max,*i*,_ corresponding to the maximum loss of relaxation *i*. In terms of the fit parameters, τ_max,*i*_ (which we will refer to as τ*_i_* in the following, for simplicity) is given by the following:
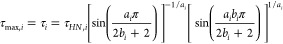
2

The shape parameters *a* and *b* can vary between 0 and 1. Specific cases of the HN function are the Cole–Cole^34^ and Cole–Davidson^35^ functions, which are obtained for *b* = 1 and *a* = 1, respectively. In the case of the Cole–Cole function, eq 2 reduces to τ*_i_* = τ_HN,*i*_. Throughout the text, we refer to τ_max,*i*_ simply as the relaxation time, and use for it the symbols τ or τ*_i_* to simplify the notation. Most dielectric spectra displayed only two relaxations in the accessible frequency window, namely, either the α and β relaxations (near and above *T*_g_) or else the intramolecular γ and γ′ relaxations (well below *T*_g_, see Section 3.3.3), so that our fit procedure only involved at most two HN functions at the time. The (primary) α relaxation turned out to be well described by a Cole–Davidson function, while all secondary relaxations could be fitted with Cole–Cole functions. This reduced significantly the actual number of free fit parameters that had to be employed in each fit.

## 3 Results

### 3.1 Differential Scanning Calorimetry Results

Figure 1 shows the DSC traces obtained for the three diazepines DIA, NOR, and TETRA. In all three cases, the as-received powders were completely crystalline, as the first heating ramp only displayed a melting endotherm with onsets at 404.1, 487.0, and 415.6 K for DIA, NOR, and TETRA, respectively. Values coincide within the experimental error with those available in the scientific literature.^24−27,36,37^ The melting point of NOR and the enthalpy of melting are both significantly higher than that of the other two derivatives, likely due to the presence of N-H···O=C hydrogen bonds, which can only form in demethylated derivative (see the next section).

The subsequent cooling ramp leads to a glassy phase for all three pharmaceuticals, and on reheating, a step-like transition can be observed in the DSC traces, corresponding to the glass transition temperature (*T*_g_). In most cases, though not in all DSC runs, TETRA and NOR displayed (at least partial) recrystallization of the supercooled liquid in the heat up run, followed again by the melting peak (see inset to Figure 1b). The recrystallized phase is the same as the initial one, as the melting temperature is the same on heating the recrystallized sample. The supercooled TETRA and NOR liquids were observed to crystallize also in dielectric spectroscopy experiments (see Section 3.3.3), while recrystallization of DIA was absent also in this case. The sample geometry and the vessel are quite different in DSC (droplet in aluminum pan) and dielectric (film in stainless steel cylinder with silica spacers) experiments. The fact that the three samples displayed the same tendency toward recrystallization under such different experimental conditions indicates that the recrystallization of TETRA and NOR probably took place by homogeneous (rather than heterogeneous) nucleation of the crystal phase. The characteristic onset temperatures of the glass transition, recrystallization, and melting points are listed in Table 1 for all three pharmaceutically active compounds, together with the melting enthalpies. The recrystallization temperature is only listed for completeness, as it did not always occur in all DSC scans at the same temperature. This is not surprising, as nucleation is a stochastic event that depends on the characteristics of the sample (heterogeneous vs homogeneous nucleation) and its history (e.g., cooling rate from the liquid phase, temperature at which it is then kept).

**Table 1:** Glass Transition (*T*_g_), Melting (*T*_m_), and Crystallization (*T*_c_) Temperatures for the Three Compounds^a^

compound	*M*_w_ (g mol^–1^)	*T*_g_ (K)	*T*_m_ (K)	*T*_c_ (K)	Δ*H*_m_ (kJ mol^–1^)
Diazepam	284.7	317.8 ± 0.4	404.0 ± 0.3		26.5 ± 1.1
			405.0 ± 0.4^27^		25.78 ± 0.19^26^
			404.5 ± 0.1^26^		
			404–406^37^		
Nordazepam	270.7	346.8 ± 1.2	487 ± 2	457 ± 1	32.5 ± 0.9
			∼489^37^		
Tetrazepam	288.8	313.0 ± 2.0	415.6 ± 1.2	385 ± 2	25.6 ± 1.3
			417^36^		

a Crystallization temperature varied from one DSC scan to the other; the reported values correspond to those of Figure 1. Melting enthalpies (ΔH**_m_**) and molecular weight (M**_w_**) are also listed, together with melting points from the previous work.

It may be seen that *T*_m_ and *T*_g_ roughly scale with one another: the *T*_g_/*T*_m_ ratio is 0.78 for DIA, 0.71 for NOR, and 0.75 for TETRA. The values for TETRA and DIA are quite similar, albeit *T*_m_ is slightly higher for TETRA than that for DIA, while *T*_g_ is somewhat lower for TETRA than that for DIA. The glass transition temperature is often found to display a correlation with the molecular weight *M*_w_. In particular, the empirical rule *T*_g_ ≈ *M*_w_^1/2^ appears to be fulfilled in the case of van der Waals molecular liquids.^38^ Such correlation probably reflects the fact that the extent of van der Waals interactions increases with the molecular mass (due to the increase of molecular polarizability and of the closest intermolecular contacts), and the fact that, at a given fixed temperature, a massive molecule has lower mobility, but it does not take into account hydrogen bonding or any other type of directional intermolecular bonds. In fact, the glass transition temperature of the studied diazepines does not correlate with the molecular weight: NOR, which has the lowest weight, has the highest glass transition temperature. The origin of the higher *T*_g_ is likely the same as that of the higher *T*_m_, namely, the presence of intermolecular H-bonds in the liquid phase of NOR. Indeed, in the absence of any H bonding the aforementioned correlation of molecular weight and glass transition temperature would result in a *T*_g_ value of NOR closer to those of DIA and TETRA, which is not observed.

### 3.2 X-ray Diffraction Results and Analysis

All three compounds display, in the crystalline phase, the same monoclinic space group (P 2_1_/c). The diazepine ring of all molecules adopts a bent boat-like conformation, with two possible isoenergetic conformers, which are mirror images of one another. The two conformers have opposite chirality and are named P (plus) or M (minus) according to the sign of the (O=)C–C(H_2_)–N=C torsion angle (see the inset to Figure 7). All three crystals contain a 1:1 ratio of P and M conformers. The geometry of the conformers is similar in all three compounds. For example, the angle formed by the C=N bond with the plane of the fused benzene ring is equal to 41.6, 38.5, and 48.6° in crystalline DIA, NOR, and TETRA, respectively.

The analysis of the X-ray structures at room temperature shows unambiguously that NOR is the only compound of the three related drugs studied that forms strong hydrogen bonds in the crystalline state, namely, intermolecular N–H···O bonds involving the amine nitrogen of the diazepine ring and the carbonyl oxygen of the same group of a nearest-neighbor molecule in the crystal structure (see Table 2). This is in agreement with the higher melting point and enthalpy of fusion of NOR compared with the other two compounds (Table 1). It is interesting to point out in this respect that while in both crystalline DIA and TETRA the carbonyl group and the adjacent methyl group are basically coplanar, with a H_3_C–N–C=O torsion angle smaller than 2°, in the case of NOR, which is *a priori* the only compound where the corresponding (peptide) moiety is expected to be planar due to the amide electronic resonance, the H–N–C=O torsion angle is instead approximately 10°. Non-planar peptide bonds are not uncommon in H-bonded structures such as proteins in their native state.^39^ In the case of crystalline NOR, the lack of planarity of the amide group is likely a consequence of H-bond formation.

**Table 2:** Shortest Intermolecular Contacts Involving a Single Hydrogen Atom (H···X, with X = O or N) and Corresponding Distance *d*, Mass Density ρ, and Hirshfeld Parameters (Volume *V*_H_, Surface *A*_H_, and Volume Normalized to Molecular Mass), for DIA, NOR, and TETRA in the Crystal Structures at Room Temperature^a^

compound	H···X	*d* [Å]	ρ [g cm^–3^]	*V*_H_ [Å^3^]	*V*_H_/M_w_ [Å^3^/a.m.u.]	*A*_H_ [Å^2^]
Diazepam	H24···O1	2.44	1.395	332.4	1.168	305.9
	H22···O1	2.53				
	H13···N2	2.81				
	H15···N2	2.79				
Nordazepam	H3···O1	2.65	1.432	308.8	1.141	287.2
	H11···O1	2.03				
	H8···N1	2.79				
	H10···N2	2.79				
Tetrazepam	H2B···O1	2.51	1.319	357.2	1.237	315.9
	H7···O1	2.61				
	H10A···O1	2.75				
	H8···N2	2.70				

a Hydrogen-atom distances *d* are reproduced from ref (40).

A recent work by some of us has shown that DIA and TETRA, while not forming N–H···O bonds, display weak but extensive C–H···O interactions between the electron-rich carbonyl group and the weakly polar C–H bonds of CH_2_ groups.^40^ While intermolecular N–H···O bonds are at least partially present also in the amorphous state of NOR, as testified by its much higher glass transition temperature (see Section 3.2), it is unlikely that the C–H···O interactions play any role in the amorphous state of the three compounds, as we argue further in Section 3.3.2.

**Figure 2:**
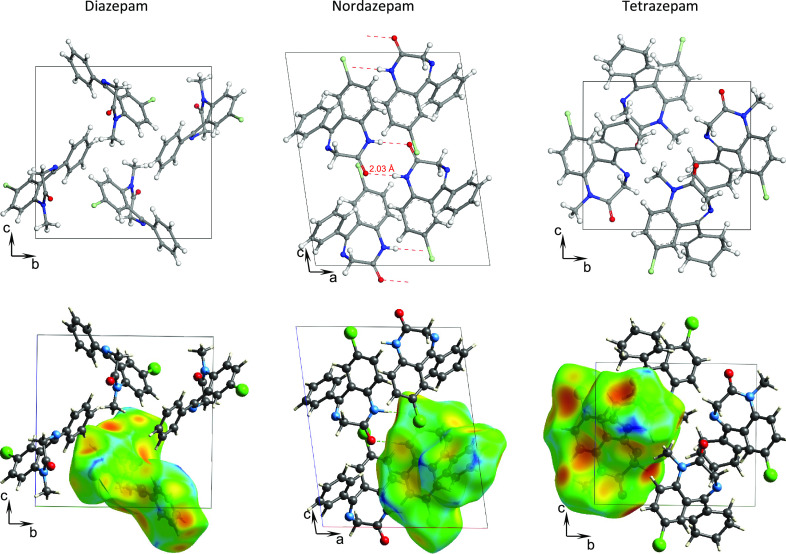
Crystal structures at room temperature of Diazepam (left, *ab* plane), Nordazepam (middle, *ac* plane), and Tetrazepam (right, *ab* plane). The projection of the unit cell is marked in light grey. N–H···O hydrogen bonds are shown for the case of Nordazepam in dashed red lines, and the corresponding intramolecular distances is indicated. Bottom: Hirshfeld surfaces of an individual molecule in the cell. Red and blue portions of the surface indicate short and long intermolecular contacts, respectively. The structure of Tetrazepam is taken from ref (40).

A straightforward comparison of the hydrogen bond scheme in the solid state of the three compounds can be carried out based on the analysis of the Hirshfeld surface areas (see Figure 2). This surface represents a particular way of partitioning the overall electron density in a molecular crystal into individual molecular units,^41^ which provides a three-dimensional image of the close contacts in the crystal by guaranteeing maximum proximity of the corresponding Hirshfeld volumes of nearest-neighbor molecules.^41−43^ The color code employed by convention is that a yellow or red color indicates points of short intermolecular contact, while blue indicates regions of the Hirshfeld surface corresponding to directions in which the intermolecular distance is comparatively longer.

**Figure 3:**
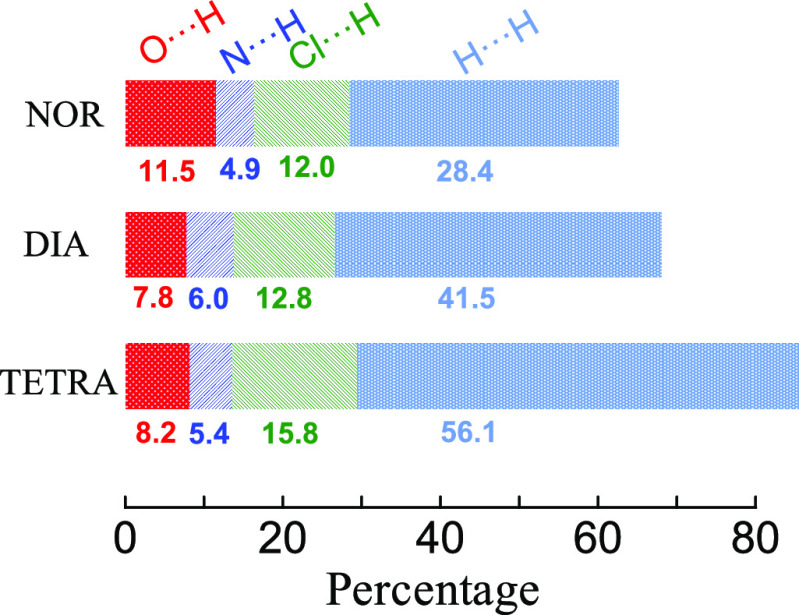
Contributions (in percentage) to the Hirshfeld surface areas of relevant intermolecular contacts: O···H, N···H, and Cl···H as well as H···H to have a reference for TETRA, DIA, and NOR compounds in the crystalline phase at room temperature as derived from the Hirshfeld surface area analysis (after ref (40)).

Figure 3, adapted from ref (40), shows the key intermolecular contacts derived from the Hirshfeld surface area analysis at room temperature in the crystalline state. It evidences the relevance of the hydrogen bond scheme for these compounds and, in particular, that of the O···H for NOR compared to DIA and TETRA, in agreement with the role of the strong N–H···O H–bond interaction in NOR.

It is interesting to note that there is a correlation between melting point, density, and Hirshfeld surface and volume parameters (Table 2). In particular, the Hirshfeld molecular volume and surface and the Hirshfeld volume normalized to molecular weight are the largest for TETRA, which has the smallest density and the lowest *T*_m_ of the three derivatives, and they are the smallest for NOR, which has the largest density and highest *T*_m_. This correlation evidences the influence on the melting temperature of the hydrogen bonds in crystalline NOR.

We point out that the correlation is instead not strictly verified when considering the glass transition temperature of all derivatives, as *T*_g,DIA_ > *T*_g,TETRA_. However, as mentioned, the *T*_g_ of NOR is significantly higher than that of the other two compounds, which is indicative of the presence of some H bonding also in the liquid phase of this compound. Instead of tightly bound stable H-bonded dimers in the liquid phase, only short-lived H bonds are expected to occur, and it is likely that a given NOR molecule only takes part, at most, in one H-bond at a time.

### 3.3 Broadband Dielectric Spectroscopy Results

**Figure 4:**
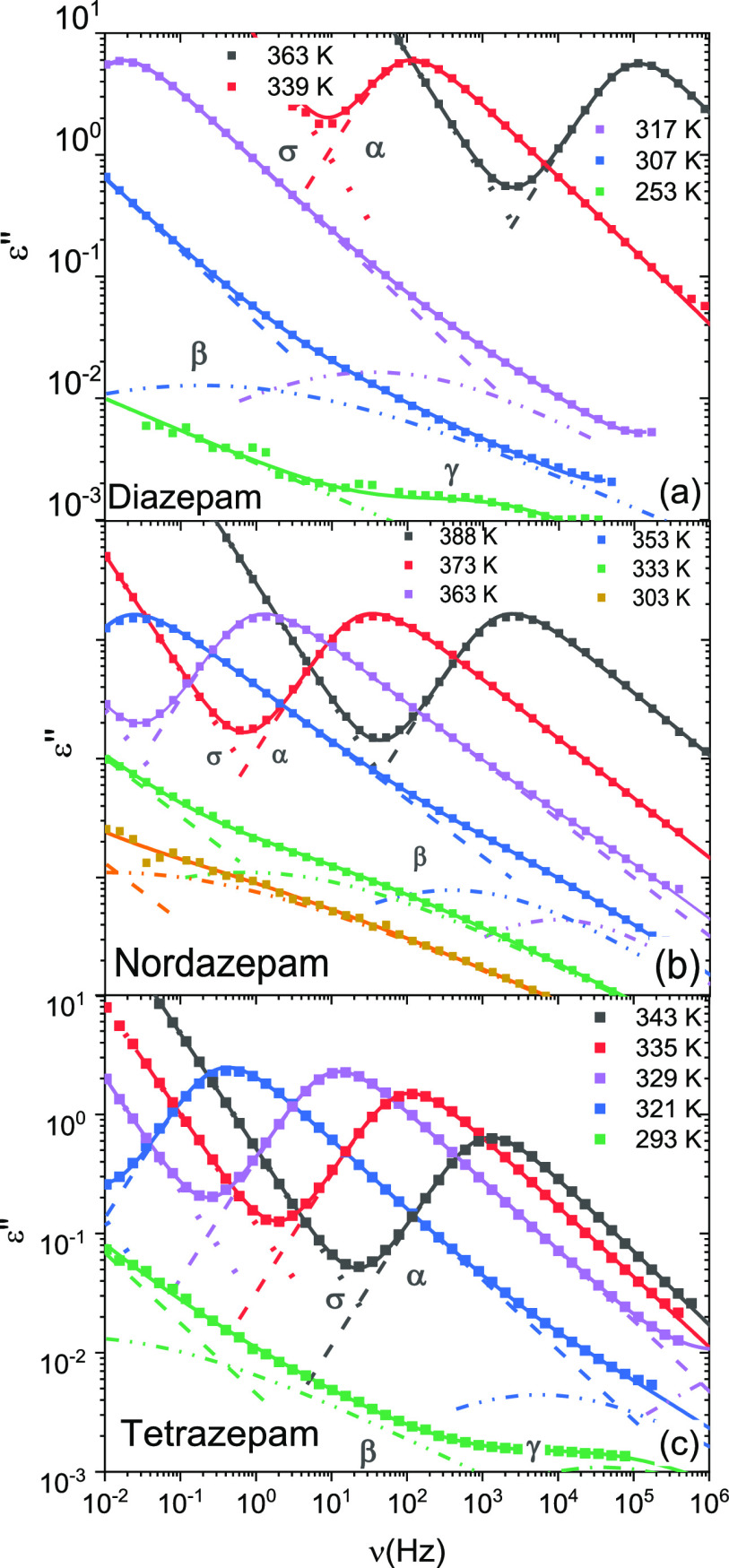
Loss spectra of amorphous DIA (a), NOR (b), and TETRA (c) (markers) at selected temperatures, as indicated in the legends, and their fits (continuous lines) as the sum of several Havriliak–Negami functions (dashed-dotted lines) and a DC conductivity background (dotted lines).

In order to see in detail how the small difference in molecular formula as well as the relevance of the hydrogen-bond network between the three studied benzodiazepines affects the molecular mobility and conformational dynamics in the amorphous state, we carried out dielectric spectroscopy experiments on all three compounds in their amorphous states. Figure 4 shows the dielectric loss function of the three compounds at few selected temperatures, plotted against the frequency of the applied electric field.

#### 3.3.1 Structural Relaxation

For all three diazepines, the most intense loss peak is observed at high temperatures (Figure 4), and corresponds to the structural relaxation (or α relaxation) of the supercooled liquid phase. Below the calorimetric glass transition temperature *T*_g_ (at which τ_α_ = 10^2^ s), the peak frequency of the α relaxation lies outside the experimental frequency window, and only the tail of the α peak is observed. When the temperature is increased above *T*_g_, the onset of the cooperative relaxation dynamics of the liquid phase is signaled by the appearance in the experimental frequency window of the α peak maximum, which then shifts to higher frequencies as the temperature is further increased.

The intensity of the α loss feature of both DIA and NOR is roughly constant above *T*_g_. Instead, recrystallization upon heating can be clearly discerned in the series of loss spectra in the case of TETRA. Indeed, at temperatures higher than 335 K the dielectric intensity of the α peak of TETRA is observed to decrease further and further as the amorphous fraction in the sample decreases (the dielectric loss intensity is proportional to the number density of molecules in the amorphous supercooled liquid state^44^).

To analyze the relaxation dynamics of the cooperative α relaxation in detail, we fitted all dielectric spectra as the sum of several Havriliak–Negami components (see eq 1), each corresponding to a distinct relaxation, in order to extract the temperature-dependent relaxation times (eq 2, see the Materials and Methods section). The fits are shown in Figure 4 along with experimental data. We found in particular that the fit with Havriliak–Negami curves resulted in a Cole–Davidson function for the structural relaxation.

**Figure 5:**
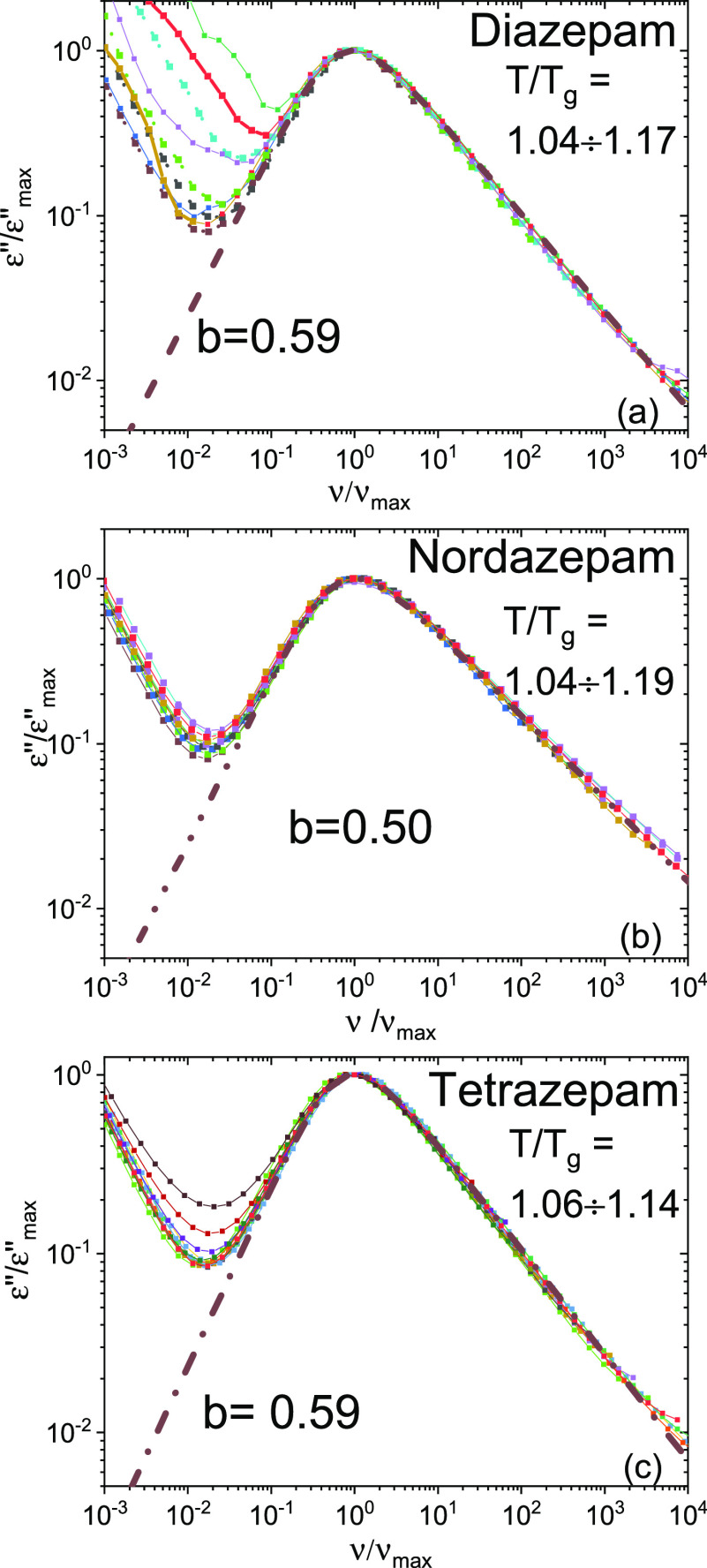
BDS spectra of supercooled liquid DIA (a), NOR (b), and TETRA (c), rescaled to the maximum of the α peak (both in intensity and in frequency). The dashed lines are Cole–Davidson (CD) fits of the α relaxation component with the indicated CD exponent *b*.

It can be observed in Figure 5 that the α peak of each compound has exactly the same shape regardless of temperature: the isothermal spectra at various temperatures could be superposed onto one another by rescaling the frequency scale and the signal intensity to those of the loss maximum. This master-curve scaling was employed in the fitting procedure, by imposing the same Cole–Davidson (CD) exponent in all high-temperature spectra of a given compound, as indicated for selected temperatures in the three panels of Figure 5. The CD exponent that best described the α peaks was found to be *b* = 0.59 ± 0.03 for DIA and TETRA, and *b* = 0.50 ± 0.02 for NOR. This result indicates a slightly greater cooperativity for NOR with respect to DIA and TETRA,^45,46^ possibly related to the presence of intermolecular H-bonds in NOR.

**Figure 6:**
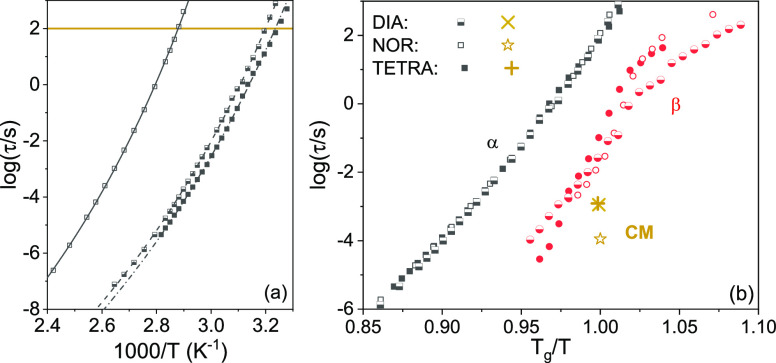
(a) Relaxation times, plotted against the reciprocal of the temperature, of the structural (α) relaxation times of the three studied diazepines. The solid line is a VFT fit of the α relaxation, and the horizontal (yellow) line marks log_10_ (τ/[s]) = 2. (b) Angell plot of the relaxation times of the α (squares) and β (circles) relaxations, together with the Johari–Goldstein relaxation times predicted at *T*_g_ using the coupling model (stars and crosses, see Section 3.3.2 for details), for all three compounds.

Figure 6 shows the α relaxation times of all three studied diazepines versus the inverse temperature (Arrhenius plot). The α relaxation time follows the Vogel–Fulcher–Tamman temperature-dependence typical of cooperative structural relaxations:^47−49^
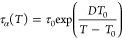
3Here, τ_0_ is the characteristic time at infinite temperature, *D* is the fragility strength coefficient, and *T*_0_ is the Vogel–Fulcher temperature. The so-called “kinetic” or “dielectric” glass transition temperature *T*_g_ of the sample is defined as the temperature at which relaxation times reaches 100 s, i.e., where log_10_(τ_α_/[s]) = 2 (horizontal yellow line in Figure 6a). The kinetic glass transition temperatures are 312.6, 309.0, and 347.2 K for DIA, TETRA, and NOR, respectively (Table 3). These values are very similar to the ones found in DSC (see Table 1), as expected.

**Table 3:** BDS Glass Transition Temperature, α-Relaxation VFT Fit Parameters, Fragility and Activation Energy of the Structural (α) Relaxation at *T*_g_, and Activation Energies of the Secondary Relaxations (β, γ and γ′), for All Three Benzodiazepines Studied

	*T*_g_ (K)	log(τ_0_/[s])	*D*	*T*_0_ (K)	
DIA	312.6 ± 0.2	–21.0 ± 0.4	10.5 ± 0.6	214 ± 3	
NOR	347.2 ± 0.2	–21.0 ± 1.0	10.3 ± 0.8	239 ± 4	
TETRA	309.0 ± 0.5	–20.7 ± 0.6	11.0 ± 2.0	207 ± 7	

	m_p_	Ea_α_ at *T*_g_ (kJ/mol)	Ea_β_ below *T*_g_ (kJ/mol)	Ea_γ_ (kJ/mol)	Ea_γ′_ (kJ/mol)
DIA	32.0 ± 1.0	(4.6 ± 0.4) × 10^2^	84 ± 6	31 ± 4	16 ± 3
NOR	32.8 ± 0.7	(4.8 ± 0.2) × 10^2^	80 ± 9	25 ± 2	7 ± 2
TETRA	31.0 ± 5.0	(4.2 ± 0.4) × 10^2^	84 ± 8	25 ± 2	11 ± 1


It is interesting to compare the dependence of the relaxation times with the inverse temperature rescaled to *T*_g_ (the so-called Angell plot), as shown in Figure 6b. The reduced temperature *T*/*T*_g_ is a measure of how far above or deep into the glass state is a sample. Remarkably, we find that the structural relaxation times of the three pharmaceuticals coincide in the Angell plot, which means that despite the structural differences and the almost 40 K of difference in *T*_g_ (and even more in *T*_m_), the supercooled liquid of these pharmaceuticals behaves cooperatively in the same way when the distance from *T*_g_ is the same. This result is reflected in the VFT parameters listed in Table 3 (in particular, in the similar value of the fragility strength coefficient *D*), and it can also be seen in the values of the so-called fragility index (*m*_p_) of the amorphous samples, which is defined as:
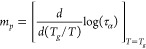
4

The fragility index is virtually the same, within the error, for DIA, NOR, and TETRA. The fragility index has often been related to the capacity of a sample to recrystallize when heated from the amorphous to the liquid state.^50−52^ This, however, is only an empirical generalization, and the present case confirms that such empirical rule fails, given the identical fragility of the three samples and their noticeable difference in recrystallization behavior. Also, the apparent activation energy at *T*_g_, i.e., the slope of the tangent to the Arrhenius plot of the structural relaxation at the glass transition, cannot be taken as a reliable predictor of the tendency toward nucleation: in fact, this parameter is again virtually identical in the case of DIA and TETRA (see Table 3), which exhibit instead very distinct nucleation tendency.

#### 3.3.2 Secondary Relaxations

Besides the α relaxation, three more secondary peaks were observed in the loss spectra at higher frequency (or lower temperature) than the cooperative loss (Figure 4), both in the supercooled liquid and the glass states. One of the secondary relaxations, which we label as β, can be observed in all three cases as a high-frequency shoulder to the structural peak. Another secondary peak (γ) is observed in the glass state of all three compounds, i.e., at low temperatures. Finally, at the lowest temperatures studied a third secondary peak (γ′) could be discerned in DIA and TETRA. In the case of NOR, the loss intensity at frequencies higher than that of the γ peak was very low, so that it would appear that the γ′ relaxation was almost absent in this compound. We have nonetheless performed a fit of this spectral region for completeness. All secondary relaxations could be fitted with symmetric Cole–Cole functions (see Materials and Methods section).

**Figure 7:**
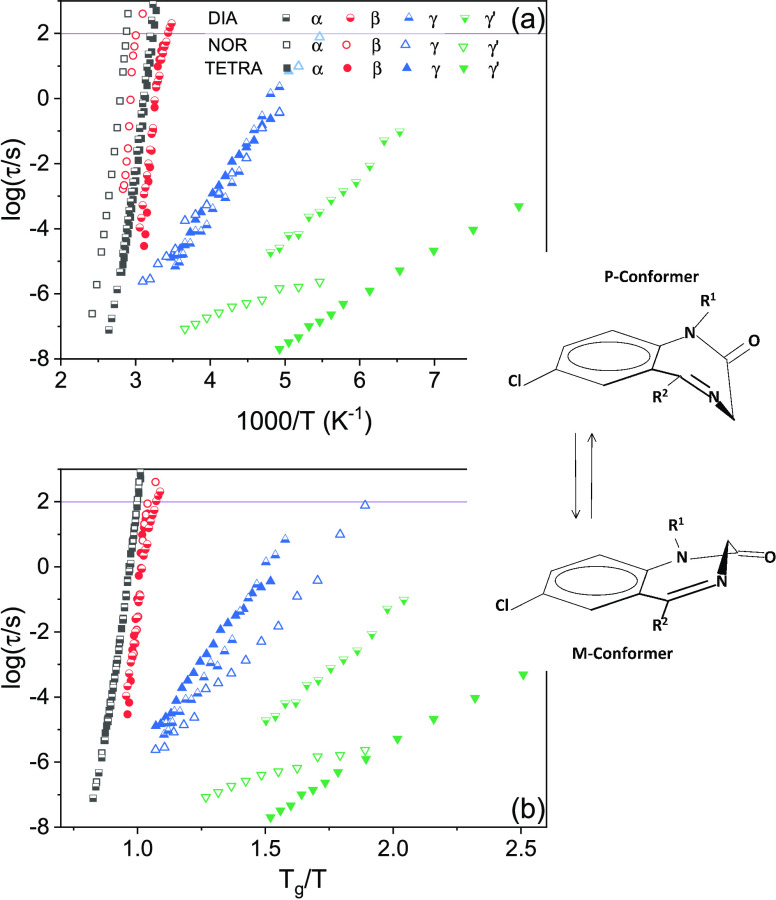
Relaxation times of all three diazepines plotted against the reciprocal of the temperature (Arrhenius plot, a) and of the reduced temperature *T*/*T*_g_ (Angell plot, b). All relaxation times are shown: α (squares), β (circles), γ (up-triangles), and γ′ (down-triangles). Inset: P (upper drawing) and M (lower drawing) conformations of the diazepine ring in the three compounds (DIA: *R*_1_ = CH_3_, *R*_2_ = phenyl; TETRA: *R*_1_ = CH_3_, *R*_2_ = cyclohexene; NOR: *R*_1_ = H, *R*_2_ = phenyl).

Figure 7a displays the full Arrhenius relaxation maps of DIA (half points), NOR (open points), and TETRA (solid points). As visible in this figure, all secondary relaxations displayed a simply activated dependence on temperature, described by the Arrhenius law:
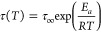
5where τ_∞_ is the characteristic time at very high (infinite) temperature (it plays the same role as τ_0_ in the VFT eq 3), *E_a_* is the activation energy, and *R* is the universal gas constant.

The β relaxation of all three compounds displayed a kink at *T* ≈ *T*_g_ (Figure 6b), where its activation energy *E*_*a,*β_ (proportional to the slope in the Arrhenius or Angell plots) was found to change discontinuously (it cannot be excluded that above *T*_g_, the activation energy of the β process is actually slightly dependent on *T*). This cross-over in the temperature dependence is typical of the so-called Johari–Goldstein (JG) secondary relaxation, a local whole-molecule relaxation that is strongly correlated with the structural one and that is a feature common to most glass formers.^53−55^

It can be easily seen in Figures 6a and 7a that the difference in glass transition temperature is reflected both in the α and β relaxations. In fact, at the same given temperature, both α and β relaxation times are much longer for NOR than for DIA or TETRA, corresponding to much slower molecular dynamics. The analysis shown in Figure 6b provides a means to further verify the JG character of the β relaxation. In fact, the β relaxations of DIA, NOR, and TETRA are observed to be virtually superposed in the Angell plot, where the three compounds all display a kink at *T*_g_/*T* ≈ 1, and the β activation energy below *T*_g_ is virtually the same (within the error) for all three compounds (see Table 3). The fact that the (secondary) β relaxation time scales with *T*_g_ (which as discussed in Section 3.3.1 is actually related to the kinetic arrest of the α relaxation) is typical of JG relaxations.^56^

The study of this type of relaxation is particularly relevant for amorphous drugs because several studies have brought forth the idea that the kinetic stability of a molecular glass is correlated with the secondary β relaxation. In particular, it has been argued experimentally that a small-molecule glass is kinetically stable only below the onset temperature of the JG relaxation, typically few tens of degrees below *T*_g_.^57^ In the case of the diazepines, the relaxation time of the β JG relaxation reaches the standard value of 100 s between 30 and 40 K below the *T*_g_ of the compound. In our experiments, NOR and TETRA displayed a tendency to recrystallize above *T*_g_, while DIA did not. It should be noted that the onset of the β relaxation is likely a minimal requirement for recrystallization: in our experiments, supercooled DIA was not observed to recrystallize during a period of few days even above the onset of the α relaxation, i.e., above *T*_g_.^58−60^

The main theoretical model concerning the JG relaxation is the Coupling Model (hereafter, CM).^61,62^ The CM interprets the JG relaxations as a local, non-cooperative whole-molecule process, which acts as the “precursor” at shorter times of the α relaxation.^61,62^ The characteristic CM relaxation times in the supercooled liquid state are given by the following approximated equation, which should approximately equal the experimental JG relaxation times:
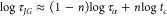
6

Here, *t*_c_ is the correlation time (usually of the order of 2 ps) and *n*, called the coupling parameter, is related to the Havriliak–Negami exponents of the α relaxation by the approximate relation^63^ 1 – *n* = (*ab*)^1/1.23^. In the case of the studied diazepines, the Havriliak–Negami function reduces to a Cole–Davidson equation with a single exponent *b*, which is found to be independent of temperature, so that the coupling parameter is constant and equal to *n* = 1 – (*b*)^1/1.23^. Equation 6 then predicts that the β relaxation time is perfectly correlated with the structural relaxation time and thus scales with *T*_g_, as indeed observed. Despite this, the relaxation times calculated with the CM theory do not coincide with the experimental JG ones. This might be due to the fact that the β relaxation is observed only as a shoulder of the α peak, in which case it has been shown that the fitting procedure that we employed does not reproduce the precursor frequency predicted by the CM. It is nevertheless worth pointing out that the difference at *T*_g_ between the theoretical times and the experimental ones can be off by as many as two orders of magnitude (see Figure 6b).

We finally discuss the fastest secondary relaxations observed in our samples. These relaxations must stem from intramolecular degrees of freedom. In the case of the benzodiazepine ring, the only degree of freedom corresponds to the chirality inversion between P and M conformers discussed in the previous section. Apart from this, all three molecules possess a torsional degree of freedom corresponding to the single covalent bond linking the fused benzodiazepine ring with the six-membered carbon ring. There are two more degrees of freedom in some of the derivatives, namely, the internal rotation of the methyl group in DIA and TETRA, and a possible conformational interconversion dynamics of the non-planar cyclohexene ring of TETRA. Neither of these processes is expected to give rise to a dielectric relaxation feature, due to the lack of dipole moment of either moiety, so that there are only two possible candidates for the experimentally observed γ and γ′ relaxations.

As visible in the Angell plot of Figure 7b, neither the γ nor the γ′ relaxation scales with the α relaxation or with the glass transition temperature, which indicates that they correspond to local relaxation processes of very low cooperativity. Looking at the relaxation maps of Figure 7a, it can be seen that the three γ relaxations have very similar relaxation times at a given fixed temperature in all three compounds and also that the corresponding activation energies *E*_a,γ_ are close for all studied diazepines (Table 3). Instead, the α and β relaxations have very different relaxation times between NOR on one hand and DIA and TETRA on the other, as stated previously, and the γ′ relaxation is quite separated in DIA and TETRA. The similarity of the γ relaxation times and activation energy, and the fact that this relaxation is unaffected by the distance from the glass transition temperature suggest that the γ relaxation is an intramolecular relaxation process common to all three diazepines.^64^

As mentioned in Section 3.2, all three studied benzodiazepines exist in two possible equivalent conformations of opposite chirality. Both conformers, P and M, are present in the crystal phase of each compound. In the gas phase and in solution, benzodiazepines are known to be relatively flexible and to display inter-conversion dynamics between the two equivalent conformations, accompanied by a reorientation by 60° of the CH_2_ moiety attached to the carbonyl group, as discussed, e.g., by Mielcarek *et al*.^65^ The conformational dynamics of DIA and NOR was reported in previous studies for molecules in solution, and it was found that the activation energy was not significantly dependent on the solvent. The conformational activation energies were found experimentally to be 74 and 52 kJ/mol for DIA and NOR, respectively.^66,67^

Because the conformational transition is accompanied also by a change in position of the polar carbonyl group and of the nitrogen atoms^67^ and thus of the direction of the molecular dipole moment, such conformational change should be observable in dielectric spectroscopy. The fact that the γ relaxation is observed in all three compounds at very similar relaxation times leads us to assign this process to the inter-conversion dynamics between P and M conformations (see inset to Figure 7). It can instead be ruled out that the γ′ relaxation can correspond to such dynamics, considering that the DIA and NOR derivatives, which have identical fused benzodiazepine rings, have γ′ relaxation times differing by more than two orders of magnitude.

It may seem surprising that the M–P interconversion takes place also in the liquid phase of NOR due to the presence of hydrogen bonds. It must however be considered that the H-bond network in a liquid phase is dynamic and in general only involves a fraction of the molecules at a given time. The dielectric signal of the P–M interconversion dynamics of NOR, namely, the γ relaxation of this compound, likely stems from the fraction of molecules that are not involved in H-bonding at a given time. It is worth pointing out, in this respect, that the relaxation time and activation energies are similar but not identical in the three compounds. We also remark that the experimental values of the corresponding activation energy in solution are roughly twice those of the γ relaxations reported in Table 3. It should however be kept in mind that the extent of H bonding will differ depending on the liquid phase, and, more importantly, our measurements of the γ dynamics are all in the glass state of the pure compound. It is well-known that the temperature dependence of the structural and JG relaxations displays an abrupt change at *T*_g_ due to the loss of ergodic equilibrium when going from the supercooled liquid to the glass phase. This is clearly visible for the case of the β_JG_ relaxation of benzodiazepine in Figure 6b, as discussed earlier. The same effect is expected to be visible for any relaxation process whose characteristic time is affected by the viscosity, and it could be that the interconversion rate between P and M conformers (γ relaxation time) is partially affected by changes of macroscopic properties of the sample such as its viscosity (although it cannot depend only on it, as Figure 7b shows). Dielectric relaxation studies of flexible heterocyclic molecules are relatively uncommon, and, to the best of our knowledge, ours is one of the few dielectric spectroscopy studies that have provided a clear identification of the ring conformational dynamics in polycyclic molecules.^28,68,69^

Finally, concerning the γ′ relaxation, both the range of temperature in which it is observed and its characteristic relaxation time are very different between DIA and TETRA, as mentioned, albeit that its activation energy is of the same order of magnitude in both compounds. Given that this relaxation is virtually absent in NOR, it is likely that it is suppressed or at least strongly hindered by the presence of intermolecular hydrogen bonds. All three studied benzodiazepines have, as mentioned, a further degree of freedom, corresponding to the torsional rotation around the covalent bond linking the fused double ring with the six-membered carbon ring.^24,65^ While the latter has basically no dipole moment, a rotation of the double ring about this covalent bond could lead to a rigid rotation of the molecular dipole moment, which would contribute a dielectric loss signal. Therefore, we tentatively assign the γ′ relaxation to the rigid rotation, likely by a small angle, of the double ring about its bond with the six-membered carbon ring. Such rotation might be partially hindered, in the case of NOR, by the presence of a network of intermolecular hydrogen bonds, which rationalizes the extremely weak signal of the γ′ relaxation in this compound. The difference between the γ′ activation energy and relaxation times of DIA and TETRA might then be attributed to the different steric hindrance of the two distinct six-member rings, namely, a bulkier phenyl ring in the case of DIA and a non-planar cyclohexene ring in the case of TETRA. This tentative interpretation is consistent with the much faster γ′ relaxation dynamics in TETRA.

#### 3.3.3 Crystallization Kinetics

Dielectric spectroscopy was employed to determine the kinetics of isothermal recrystallization from the supercooled liquid state of NOR and TETRA (as mentioned, DIA was not observed to recrystallize in short times). To this purpose, we acquired series of dielectric spectra at fixed temperature and analyzed the variation in time of the static dielectric constant, which is related to the dielectric intensity of the structural relaxation process. Since NOR has significantly higher glass transition temperature than TETRA, at temperatures at which the latter compound showed recrystallization at detectable rates, NOR is close to being in the glass state, where the recrystallization onset time and recrystallization rates are too long to allow a dielectric measurement. Therefore, because such “isothermal comparison” of the recrystallization process cannot be carried out, we have chosen different temperatures to study recrystallization at roughly the same reduced temperature *T*/*T*_g_.

**Figure 8:**
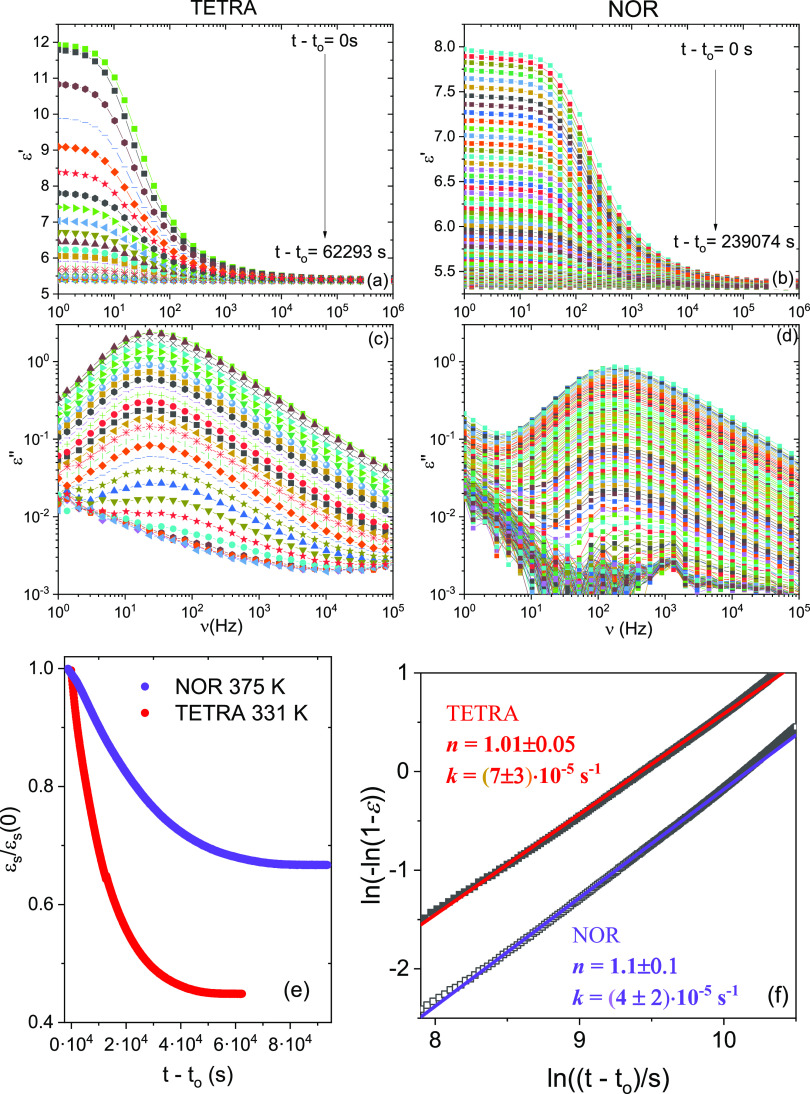
Real and imaginary permittivity spectra of TETRA (a, c) and NOR (b, d) at *T* = 331 K and *T* = 375 K, respectively, acquired at different times during recrystallization of the supercooled liquid sample. (e) Time-dependence of the static permittivity ε_s_, taken as the value of ε′(*f*) at the frequency of 1 Hz for TETRA and 2 Hz for NOR. (f) Avrami plot of the recrystallization data. Markers are experimental points and continuous lines are fits with the Avrami eq 8.

Figure 8 displays the series of isothermal permittivity spectra (real and imaginary part) during recrystallization of TETRA at *T* = 331 K (corresponding to *T*/*T*_g,TETRA_ = 1.07) and of NOR at *T* = 375 K (corresponding to *T*/*T*_g,NOR_ = 1.08). The effect of recrystallization is visible as a decrease over time of the dielectric intensity of the α loss feature, or equivalently a decrease of the static permittivity value ε_s_, defined as the value of ε′(*f*) at the lowest frequency displayed in the figure (*f* = 1 Hz for TETRA and *f* = 2 Hz for NOR, respectively). The onset time *t*_o_ of the recrystallization process was determined as the time at which the initially constant value of ε_s_ in the supercooled liquid phase was observed to start decreasing. The evolution of ε_s_ with time elapsed from the start of the recrystallization is displayed in Figure 8e. It is clear that the recrystallization of NOR at *T*/*T*_g,NOR_ = 1.08 is slower than that of TETRA at *T*/*T*_g,TETRA_ = 1.07, despite the fact that the structural (α) relaxation frequency and thus the cooperative mobility are, under such conditions, higher by a factor of four in NOR than in TETRA, as testified by the position of the loss maxima in panels (c) and (d) of Figure 8.

In order to study the kinetics of recrystallization, we define as customary^9,72^ a normalized static permittivity value as:
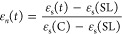
7

Here, ε_s_(SL) and ε_s_(C) are the static permittivity of the supercooled liquid and the crystal phase, as measured before the onset of nucleation of the crystal phase and at the end of the crystal growth, respectively, while ε_s_(*t*) is the static permittivity of the partially recrystallized sample as function of time. The global kinetics of crystallization can be modeled with the help of the Avrami equation,^70,71^ which is based on the nucleation-and-growth model of the transition from the liquid to the crystal phase. According to this model, the renormalized static permittivity should vary in time as:^72,73^
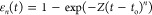
8Here, *n* is the Avrami exponent and *Z* is a constant from which the recrystallization rate in s^–1^ can be obtained^9,74^ as *k* = *Z*^1/*n*^. According to eq 8, the quantity ln(−ln(1 – ε_n_)) should be linearly proportional to the logarithm of the time elapsed since the onset of recrystallization, *t* – *t*_o_. This is indeed observed in the Avrami plot displayed in Figure 8f.

The values of the obtained fit parameters are *n* = 1.01 ± 0.05, *k* = (7 ± 3)·10^–5^ s^–1^ for TETRA and *n* = 1.1 ± 0.1, *k* = (4 ± 2)·10^–5^ s^–1^ for NOR. The fact that the value of the Avrami exponent is close to unity for both derivatives indicates a strongly anisotropic (one-dimensional) growth of the crystal phase after a sporadic nucleation event.^19,75,76^ A value of *n* = 1 also allows direct estimation of the crystal growth rate, that is, separation of the nucleation and crystal growth phases of the recrystallization.^76^ The vertical separation in Figure 8f, in which assuming an identical value of *n* can be related to the difference in recrystallization rate *k* between the two samples (see the discussion of Figure 6 of ref (75), confirms the slower crystal growth kinetics directly visible in Figure 8e, and is consistent with the experimental ranges of values of the recrystallization rate *k* of TETRA and NOR under these conditions.

We also studied the recrystallization of NOR at *T* = 368 K (*T/T*_g_ = 1.06). The latter temperature was chosen so that the structural relaxation frequency was the same for both compounds (a condition usually referred to as “isochronal condition” in the scientific literature). Because the two compounds have similar fragility indexes, this condition is very similar to that of same reduced temperature, *T*/*T*_g_. The crystal growth rate of NOR was so slow under these conditions (at a temperature only 5 K below the crystallization temperature of Figure 8) that we could not complete it during three full days of continuous measurements. The crystallization (growth) rate *k* for NOR at 368 K (*k* = (7 ± 3)·10^–6^ s^–1^) was one order of magnitude smaller than that for TETRA at 331 K, and our experiments show that the (homogeneous) nucleation time is very different in DIA with respect to its derivatives.

## 4 Discussion

These results on three very similar molecules have important implications. Several recent studies on different glass former compounds have reported that the crystallization time (or equivalently the inverse crystallization rate) and the structural relaxation time are correlated with one another.^59,76,77^ These studies have shown that there is a power-law correlation between the recrystallization time and τ_α_. Our study of very similar molecular derivatives shows, in a very direct way, that there cannot be a general quantitative relation between the *absolute numerical values* of these two quantities in different samples. This is not surprising in view of the fact that different compounds have, in general, different power law exponents;^76,77^ our study further shows that even related molecular derivatives have different correlation laws. Hence, the correlation between τ_α_ and the crystallization growth rate is not only limited to a temperature interval, as implied by the standard model of crystallization by nucleation and growth and as shown experimentally in a recent study of ours^9^ but also it cannot be used as an *a priori* predictor of crystallization tendency or rate. Indeed, our study confirms that supercooled liquids of very similar glass-former molecules have, at the same value of τ_α_, not only very different nucleation times but also quite distinct crystal growth rates, depending, in the present case, on the extent of hydrogen bonding. These results are in agreement with the standard model of crystallization by nucleation and growth: in fact, the nucleation step is mainly determined by the difference between bulk free energy and by the interfacial tension of the liquid and crystalline phases, rather than the molecular mobility; and similarly, the growth kinetics of crystalline nuclei is not uniquely determined by the molecular mobility alone. Our findings imply that, to further improve our experimental understanding of the kinetic stability of amorphous pharmaceutics, correlations with other (possibly macroscopic) quantities, related to the local structure in the liquid and crystal states, should be investigated, beyond that with the structural mobility or viscosity.

To summarize, we have studied three diazepine derivatives of very similar mass and molecular structure (Diazepam, Nordazepam and Tetrazepam), to determine how the differences in the molecular structure and thus intermolecular interactions affect the properties of the crystalline and amorphous states of these pharmaceutical compounds. Nordazepam is the only compound that displays N–H···O hydrogen bonds, leading to the formation of H-bonded dimers in the crystalline phase, which as a consequence exhibits significantly higher melting point and melting enthalpy compared to the other two compounds, which display similar melting temperatures and enthalpies. Nordazepam has the highest density in the crystalline state and the smallest Hirshfeld surface and volume of the three. The diazepine ring has a non-planar structure, and all three benzodiazepine crystalline structures consist of two isoenergetic P and M conformers, which are mirror images of one another and occur in a 1:1 ratio. The characteristic angles of these conformations are similar in the three compounds.

The liquid phase of Nordazepam displays significantly higher glass transition temperature than the other two compounds, and the dielectric signature of the structural α relaxation is broader in this compound than in the other two, indicative of a more cooperative structural relaxation dynamics. These two experimental observations indicate at least partial hydrogen bonding also in the liquid phase of Nordazepam. The presence of different possible molecular conformations, as well as the torsional degree of freedom between the fused double ring and the six-membered carbon ring, further enrich the relaxation map in the amorphous (supercooled liquid and glass) state. All three compounds display a Johari–Goldstein β relaxation, visible as a shoulder to the main α loss feature. The relaxation time of both α and β relaxations scales with the temperature normalized to the glass transition temperature (*T*/*T*_g_). The curvature of the structural relaxation is the same in all three compounds leading to a virtually identical kinetic fragility index (*m*_p_ ≈ 32).

The three compounds display intramolecular relaxations in the glass state, one of which is common to all of them, and corresponds to the P-M inter-conformer conversion dynamics of the diazepine heterocycle. This relaxation does not scale with the cooperative molecular mobility (α relaxation time), although comparison with liquid-phase studies indicates that its activation energy is slightly lower in the glass state compared to the liquid. A fourth, high-frequency secondary relaxation is present only in Diazepam and Tetrazepam, likely associated with the rigid rotation of the fused double ring relative to the apolar six-membered ring. Its almost complete absence in Nordazepam can be rationalized by the existence of strong hydrogen bonds between the double rings of neighboring molecules, which prevents such rotation.

While supercooled liquid Tetrazepam and Nordazepam are observed to recrystallize upon heating, with Avrami exponents close to unity in both cases, Diazepam does not display any tendency toward recrystallization at least over short periods of time. The crystallization rates of Tetrazepam and Nordazepam differ, under isochronal conditions of the structural α relaxation, by more than a decade. We conclude that the kinetic stability of amorphous diazepines, and especially the nucleation tendency, does not display any correlation with the density, kinetic fragility index, or structural or secondary Johari–Goldstein relaxation time. Only the crystal growth rate, and not the tendency toward nucleation, is affected by the presence of a hydrogen-bond network. Our comparison between very similar molecular derivatives provides a direct confirmation that the search for microscopic criteria for the kinetic stability of amorphous pharmaceuticals must include, besides molecular interactions and relaxation dynamics, other parameters related to the difference in the (local) structure between the liquid and crystal phases.
