# Correction to “Comparative Physical Study of Three Pharmaceutically Active Benzodiazepine Derivatives: Crystalline versus Amorphous State and Crystallization Tendency”

**DOI:** 10.1021/acs.molpharmaceut.1c00654

**Published:** 2021-09-16

**Authors:** Sofia Valenti, Maria del Barrio, Philippe Negrier, Michela Romanini, Roberto Macovez, Josep Lluis Tamarit

An error was noticed in Table 3 of the original article after publication. In the determination of the activation energies of the dielectric relaxations, a numerical mistake was made by not properly considering that the latter are defined in terms of a natural logarithm while the experimental data are represented in a base-ten logarithmic scale. The mistake affected the activation energy of the secondary relaxations and the determination of the fragility strength coefficient *D* and kinetic fragility index *m*_p_ associated with the primary relaxation. As a consequence of the logarithmic base change, several entries of Table 3 need to be corrected by a factor ln(10). We have replaced the faulty table with the new Table 3 reported here below. With this correction, the fragility of each compound is now coherent with the effective activation energy of the primary (α) relaxation at *T*_g_, which was computed correctly in the original paper. In the abstract, we have correspondingly replaced the value of the kinetic fragility index (which was erroneously given as *m*_p_ ≈ 32, a relatively low value) with the correct value of *m*_p_ ≈ 73, which is a more common value for molecular glass formers. We have done the same replacement at the end of the Discussion section.

**Table 3:** BDS Glass Transition Temperature, α-Relaxation VFT Fit Parameters, Fragility, and Activation Energies of the Secondary Relaxations (β, γ, and γ′) for the Three Benzodiazepines

	*T*_g_ (K)	log(τ_0_/[s])	*D*	*T*_0_ (K)
DIA	312.6 ± 0.2	–21.0 ± 0.4	24.3 ± 1.4	214 ± 3
NOR	347.2 ± 0.2	–21.0 ± 1.0	23.6 ± 1.9	239 ± 4
TETRA	309.0 ± 0.5	–20.7 ± 1.0	27 ± 4	207 ± 7

	*m*_p_	Ea_**α**_ at *T*_g_ (kJ/mol)	Ea_β_ (kJ/mol)(below *T*_g_)	Ea_**γ**_ (kJ/mol)	Ea_**γ′**_ (kJ/mol)
DIA	73 ± 7	(4.6 ± 0.4) × 10^2^	(1.9 ± 0.1) × 10^2^	72 ± 9	37 ± 7
NOR	73 ± 9	(4.8 ± 0.4) × 10^2^	(1.8 ± 0.2) × 10^2^	58 ± 5	16 ± 5
TETRA	72 ± 16	(4.2 ± 0.4) × 10^2^	(1.9 ± 0.2) × 10^2^	58 ± 5	25 ± 3

This numerical correction does not change the main results and conclusions of the original article. In particular, it remains true that the fragility indices are virtually identical in all three diazepine derivatives, and therefore that this parameter cannot be employed as a reliable predictor of the crystallization tendency.

It is worthwhile to point out that this numerical correction further corroborates one important conclusion of our work, namely, the molecular interpretation of the secondary γ relaxation process. In fact, the correct activation energies of the γ relaxations of diazepam and of its nordazepam derivative are 72 ± 9 and 58 ± 5 kJ/mol, respectively, which are now compatible with those found in previous studies by NMR experiments and *ab initio* calculations, where the conformational ring-inversion activation energies were reported to be 74 and 52 kJ/mol^1^ and in the ranges 72.4–74.1 and 44.8–47.3 kJ/mol,^2^ respectively, for the two compounds. This agreement confirms the assignment of the γ relaxation to the diazepine ring-inversion dynamics. On page 1827, right column, we have correspondingly modified the sentence in which we reported the discrepancy between the activation energy of the γ relaxations of Table 3 with those of previous studies in solution, and removed the rationalization that we had given in terms of a possible difference between the liquid and glassy state. As a matter of fact, our correction allows reaching the further, interesting conclusion that the activated behavior of the ring-inversion relaxation of liquid diazepines is not affected by the transition to the glass state.

We thank Prof. H.P. Diogo from the Chemistry Engineering Department of the Universidade Técnica de Lisboa for first observing the incongruency between the activation energy and the fragility of the primary relaxation that has led us to check our results.
